# Supramolecular Polymer-Based Delayed Crosslinking Weighted Fracturing Fluid with a Double Network for Ultra-Deep Reservoirs

**DOI:** 10.3390/gels12050368

**Published:** 2026-04-28

**Authors:** Shenglong Shi, Jinsheng Sun, Kaihe Lv, Jingping Liu, Taiming Zhang, Yajie Li, Xiaoshuang Chen, Kangrui Xu

**Affiliations:** 1 College of Science, Qingdao University of Technology, Qingdao 266580, China; 2 Department of Petroleum Engineering, China University of Petroleum (East China), Qingdao 266580, China; sunjsdri@cnpc.com.cn (J.S.); lkh54321@126.com (K.L.);

**Keywords:** ultra-deep reservoir, weighted fracturing fluid, low friction, delayed crosslinking, double network

## Abstract

Hydraulic fracturing in ultra-deep reservoirs faces significant challenges, including high wellbore friction and inadequate thermal stability of conventional fracturing fluids. To address these issues, we developed a potassium formate-weighted fracturing fluid with delayed crosslinking, excellent friction reduction, and superior temperature resistance, using a hydrophobic associating polymer thickener and a multi-ligand organic zirconium crosslinker. The weighted fracturing fluid has a density of 1.4 g/cm^3^ and completes crosslinking within 300 s at 90 °C. It achieves a maximum friction reduction rate of 63.2%. Below 60 °C, the system relies on a supramolecular thickener network for low viscosity and friction reduction; above 60 °C, chemical crosslinking between the thickener and zirconium ions creates a dual-network structure that significantly enhances temperature and shear resistance. After 120 min of shearing at 200 °C and 170 s^−1^, the retained viscosity reaches 75.3 mPa·s. Complete gel breaking is achieved by sodium bromate via an oxidation reaction. This dual-network delayed crosslinking system successfully reconciles the conflict between low wellbore friction and high-temperature proppant-carrying capacity. This work presents a superior weighted fracturing fluid for ultra-deep reservoirs, as well as an innovative technique for their development.

## 1. Introduction

With the deepening of oil and gas exploration and development, ultra-deep oil and gas resources (burial depth > 8000 m) have become the primary breakthrough for increasing production [1,2]. These reservoirs typically feature ultra-high temperature (>200 °C), extremely low permeability (<0.1 × 10^−3^ μm^2^), and nearly zero natural productivity [3,4]. Hydraulic fracturing is essential to create complex fracture networks that enable efficient production. However, during fracturing operations in ultra-deep wells, the low density of conventional fracturing fluids leads to insufficient hydrostatic pressure, resulting in excessively high wellhead pressure that challenges both surface pumping equipment and wellbore integrity [5,6]. Increasing fluid density is an effective way to raise hydrostatic pressure and thus reduce wellhead pressure. High-weighted fracturing fluids have been developed for this purpose [7,8].

The choice of weighting agents is critical. Inorganic salts such as sodium chloride (NaCl), potassium chloride (KCl), calcium chloride (CaCl_2_), sodium bromide (NaBr), and sodium nitrate (NaNO_3_) are the most used weighting agents for fracturing fluid [9,10]. KCl and NaCl can only achieve a density of 1.1–1.2 g/cm^3^, which limits their weighting effect [11,12]. CaCl_2_ reacts rapidly with formation fluids, forming precipitates that plug rock pores and impair fracture conductivity [13]. NaBr provides a higher density (1.4 g/cm^3^) but is expensive [14]. NaNO_3_ is restricted due to its use as a raw material in explosives [15]. In contrast, formates such as potassium formate (HCOOK) and sodium formate (HCOONa) can prepare solutions with a density of approximately 1.4 g/cm^3^ at a significantly lower cost. Moreover, formates are nearly non-corrosive and highly compatible with formation water [16,17]. However, because formate has reducing properties, it competes with the thickener for the oxidant, making it difficult to break HCOOK-weighted gels with conventional breakers like ammonium persulfate. Recent work has demonstrated that the non-free radical oxidant NaBrO_3_ can effectively break such gels [18].

Based on thickener type, weighted fracturing fluids are categorized into natural plant gum and synthetic polymer systems [19]. Guar and its derivatives are widely used due to their excellent performance, but their glucoside bonds become highly unstable above 180 °C, which is the upper limit for guar-based fluids [20,21]. Therefore, for ultra-deep and ultra-high temperature reservoirs, synthetic polymers, especially polyacrylamide-based copolymers, are increasingly preferred. To meet the demanding performance requirements, specific functional groups (e.g., strong electrolytes, ring structures, betaine, and hydrophobic groups) are introduced to enhance temperature resistance, salt tolerance, and shear stability [22,23]. For example, Yang et al. developed a HCOONa-weighted fracturing fluid using a thickener composed of acrylamide, acrylic acid, 2-acrylamide-2-methylpropane sulfonic acid, and sodium p-styrene sulfonate; the fluid operated at 180 °C, but its maximum friction reduction rate remained below 50% [24]. High-strength gel frequently performs poorly in reducing wellbore friction during ultra-deep fracturing, even though the ultra-high temperature environment necessitates a high-strength polymer network structure to guarantee system stability.

A critical issue in ultra-deep fracturing is the contradiction between “temperature resistance in the reservoir” and “friction reduction in the wellbore” [25]. High-strength gels required for ultra-high temperatures often exhibit poor friction reduction performance in the long wellbore. Delayed crosslinking technology has been introduced to resolve this conflict. By controlling the crosslinking time and rate, the fracturing fluid maintains low viscosity in the wellbore (reducing frictional resistance) and gradually builds a high-strength gel only after entering the high-temperature reservoir, thereby ensuring effective proppant transport [26]. Organic transition metal crosslinkers are considered a primary strategy for achieving such delayed crosslinking in ultra-high temperature fracturing [27]. Their crosslinking mechanism involves ligand exchange: the original ligand in the crosslinker is gradually replaced by carboxylate or other functional groups from the thickener [28]. Consequently, the crosslinking rate can be tuned by selecting appropriate organic ligands, allowing the reaction to occur primarily in the reservoir rather than in the wellbore, effectively reducing shear time and enhancing the friction reduction capability. However, this approach requires that the fracturing fluid possess sufficient viscosity and viscoelasticity before crosslinking to suspend proppants [29]. Typically, this is achieved by increasing the thickener concentration or molecular weight, which significantly raises costs and makes subsequent gel breaking more difficult, leading to unavoidable reservoir damage. Therefore, for the thickener in the weighted fracturing fluid, it is required to have good friction reduction, viscoelasticity, and low damage to the reservoir at low dosages.

Strengthening intermolecular forces offers a practical route to increase viscosity and viscoelasticity without raising thickener dosage. In recent years, hydrophobic associating polymers have attracted considerable attention. These polymers contain hydrophobic side chains that form dynamic supramolecular associations (e.g., hydrophobic interactions, hydrogen bonding, cation-π, and π-π stacking) in aqueous solution, creating a reversible physical network. Wang et al. [30] incorporated hydrophobic groups into a polymer to develop a weighted fracturing fluid for high-salt reservoirs; after 90 min of shearing at 180 °C, the viscosity remained above 50 mPa·s and density of 1.10 g/cm^3^, but the average core damage rate was approximately 30%, indicating that the fracturing fluid caused slightly more serious damage to the formation. Jian et al. [31] synthesized a zwitterionic hydrophobic polymer and combined it with a delayed crosslinker, achieving a friction reduction of more than 70% before crosslinking [32]. Nevertheless, these fluid systems were not specifically designed for ultra-deep high-temperature wells, and their delayed crosslinking time was insufficient for ultra-deep applications. Xu et al. [6] developed a new type of delayed crosslinking gel fracturing fluid using mussel-inspired thickener and organic zirconium delayed crosslinkers. Due to the existence of supramolecular interactions between hydrophobic association polymer chains, it was possible to create a dense network structure through supramolecular interactions. As a result, the supramolecular polymer system effectively suspended the proppant before crosslinking due to its strong viscoelasticity at a lower concentration at 25 °C. With rising temperature, the zirconium crosslinker induced crosslinking of the supramolecular polymer system, thereby creating a double network gel that might make up for the fracturing fluid’s loss of viscosity at ultra-high temperatures of 200 °C. Furthermore, a clean fluid would be produced following the gel breaking because of the low thickener concentration. However, the salt weighting properties of the aforementioned fracturing fluid were not mentioned.

The concept of a double crosslinked polymer network, which combines a reversible supramolecular physical network with a permanent covalent chemical network, has shown great promise for high-temperature fracturing fluids. Several recent studies have explored such double-network hydrogels for fracturing applications, but most have focused on non-weighted systems or have not addressed the specific challenges of ultra-deep reservoirs. Despite the advances summarized above, a fracturing fluid that simultaneously possesses all of the following desirable properties has not yet been reported: salt weighting capability with a density of at least 1.4 g/cm^3^, low friction characterized by a friction reduction rate exceeding 50%, a sufficiently long delayed crosslinking time of more than 200 s, excellent temperature and shear resistance that remains stable at 200 °C, and low reservoir damage. In particular, the combination of a hydrophobic associating polymer, which forms a supramolecular network at low temperature, with an organic zirconium delayed crosslinker, which forms a chemical network at high temperature, has not been systematically evaluated for HCOOK-weighted systems in the context of ultra-deep reservoir fracturing [32].

In this study, a HCOOK-weighted fracturing fluid with delayed crosslinking characteristics, good friction reduction, and excellent temperature resistance was composed of a hydrophobic association polymer thickener and a multi-ligand organic zirconium delayed crosslinker. The network structure created by supramolecular forces could arise as a result of the supramolecular interaction between thickener chains. As a result, the supramolecular thickener solution had a higher viscosity and viscoelasticity at a lower concentration, making it ideal for reducing friction and suspending the proppant before crosslinking at the surface and wellbore stages. When the temperature increased, an organic zirconium crosslinker was used to crosslink the supramolecular polymer system, forming a double network gel that could compensate for the fracturing fluid’s viscosity loss at ultra-high temperatures as it proceeded further into the reservoir. Additionally, due to the low concentration of the thickener, complete gel breaking was accomplished by using the non-free radical oxidant sodium bromate (NaBrO_3_) as a gel breaker. The application of this HCOOK-weighted fracturing fluid to ultra-high temperature fracturing operations may furnish theoretical insights and engineering support for the successful development of ultra-deep hydrocarbon resources.

## 2. Results and Discussion

### 2.1. Concentration Optimization of the Thickener and Crosslinker

**Figure 1:**
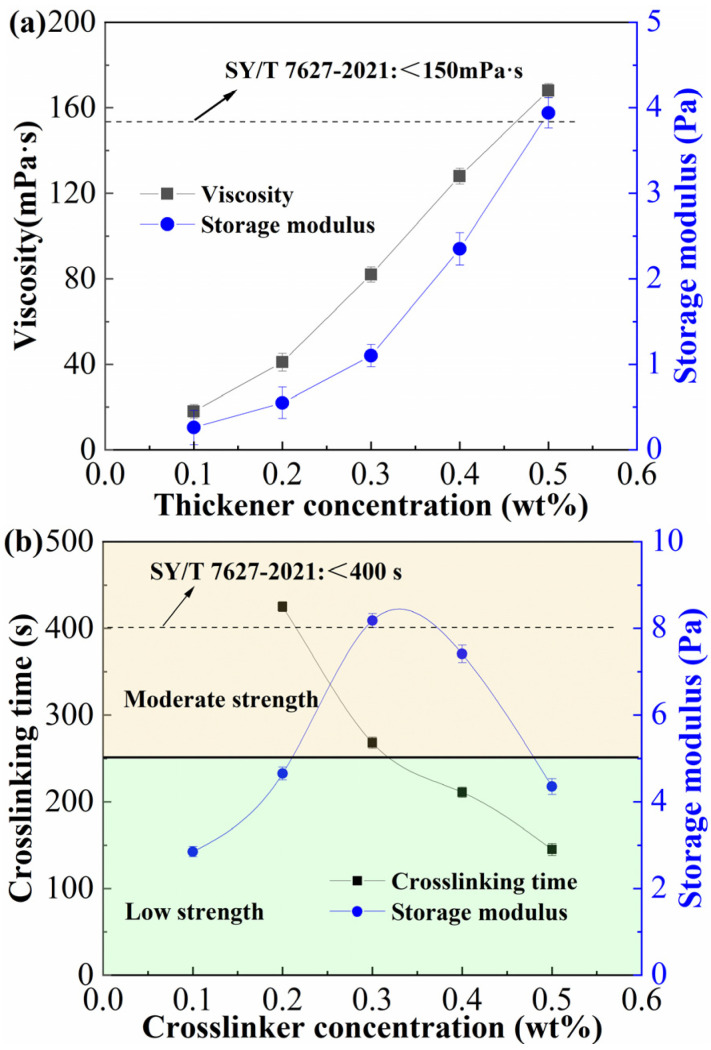
Performance characteristics of thickener fluid and weighted fracturing fluid: (**a**) viscosity and storage modulus of thickener solutions at different ASDM concentrations (0.1–0.8 wt%) measured at 25 °C and a shear rate of 170 s^−1^; storage modulus was measured at 1 Hz and 1% strain; (**b**) crosslinking time (at 90 °C) and storage modulus of weighted fracturing fluids containing 0.4 wt% ASDM and varying crosslinker concentrations (0.1–0.5 wt%).

Thickener and crosslinker are the major additives used in weighted gel fracturing fluid, and the concentrations at which these additives are utilized have a substantial impact on the performance of fracturing fluid. For the thickener optimization, ASDM concentrations tested were 0.1, 0.2, 0.3, 0.4, and 0.5 wt%, all solutions were prepared in HCOOK brine with a density of 1.4 g/cm^3^. Figure 1a illustrates the outcomes of optimizing the thickener concentration without the use of a crosslinker by examining the viscosity and storage modulus of the thickener solution. With increasing thickener concentration, the viscosity and modulus of solution increased, and none of the systems were able to gel to a hanging condition. This revealed that thickener molecules at higher concentrations became entangled, resulting in increased intermolecular interaction and a stronger thickening structure. An excessive thickener concentration would cause the viscosity to grow during fracturing, increasing pumping frictional resistance. This resistance would make the subsequent gel-breaking process more challenging and raise the total cost of fracturing. Therefore, 150 mPa·s was chosen as the recommended criterion. Based on this criterion, the appropriate thickener concentration should be less than 0.5 wt%.

The weighted gel fracturing fluid has a characteristic of temperature resistance and shear resistance due to the network-like spatial structure created by the combination of thickener and crosslinker. To provide the appropriate delayed crosslinking control, the amount of crosslinker was optimized by taking crosslinking time and crosslinking strength as performance indicators. The thickener concentration was 0.4 wt%, and the crosslinker (Zr-LSE) concentrations tested were 0.1, 0.2, 0.3, 0.4, and 0.5 wt%. The crosslinking performance of fracturing fluid was shown in Figure 1b. Figure 1b reveals that at a low crosslinker concentration of 0.1 wt% and 90 °C, no gel capable of hanging was formed. The resulting system was mechanically weak, giving a storage modulus of only 2.85 Pa. It was discovered that a hangable gel could be produced and that the crosslinking time could be changed between 150 s and 430 s when the crosslinker concentration was between 0.1 wt% and 0.5 wt%. A short crosslinking time was linked to a high crosslinker concentration. When the crosslinker concentration was 0.3 wt%, the storage modulus reached its peak and then decreased. When the crosslinker concentration was increased, more zirconium ions were released over a shorter period, leading to crosslinking that was both faster and stronger. An over-crosslinking phenomenon would occur if the crosslinker concentration were too high, which would also weaken the crosslinking [33]. As a result, there was an ideal concentration ratio between the crosslinker and thickening. Using a crosslinking time of less than 300 s and a high crosslinking strength as the standard, the optimal crosslinker concentration was 0.3 wt%.

### 2.2. Friction Reduction

**Figure 2:**
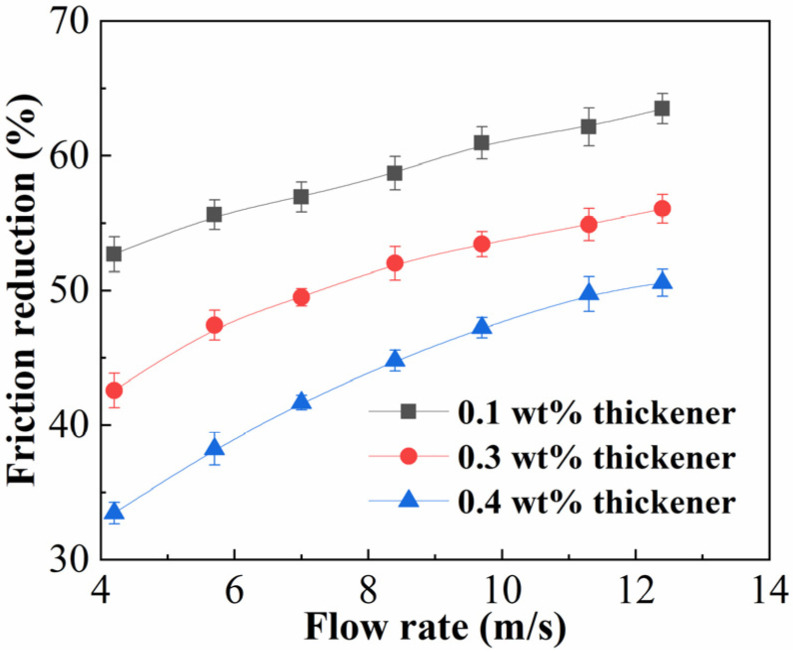
Friction reduction rate of fracturing fluids with different ASDM concentrations (0.1–0.4 wt%) as a function of flow rate.

The presence of an ultra-long wellbore during ultra-deep fracturing operations highlights the necessity of using a fracturing fluid that can minimize wellbore friction. Figure 2 displays the measured friction reduction under various fracturing fluid displacements. ASDM concentrations were 0.1, 0.3, and 0.4 wt%. With rising flow rate, the friction reduction rate increased accordingly, which eventually stabilized at a certain level. The highest friction reduction rate was 63.5% at 0.1 wt% thickener concentration. As the concentration increased, the friction reduction rate decreased continuously, and the friction reduction rate steadily dropped, reaching 50.3% when the thickener concentration was 0.4 wt%, which was higher than 50% required by the industry standard. This was due to the thickener molecules absorbing the vortex energy in the turbulence, and the resilient stability of the thickener network structure helped mitigate the negative impact of high shear rates on the friction reduction rate [12]. Increasing the flow rate caused greater turbulence in the fluid, and this turbulent action in turn pulled the thickener’s network structure into a stretched state, which served to improve the friction-reducing effect. As thickener concentration increased, so did the number of molecular chains, as well as their interactions and curling. This increased molecular chain curling made it difficult to achieve complete extension of thickener molecular chains, resulting in the consumption of a significant portion of fluid energy. Concurrently, the spatial aggregation brought on by molecular chain entanglement raised the viscosity of the solution, which in turn raised the fluid flow resistance and lowered the friction reduction rate [34].

### 2.3. Proppant-Carrying Capacity

**Figure 3:**
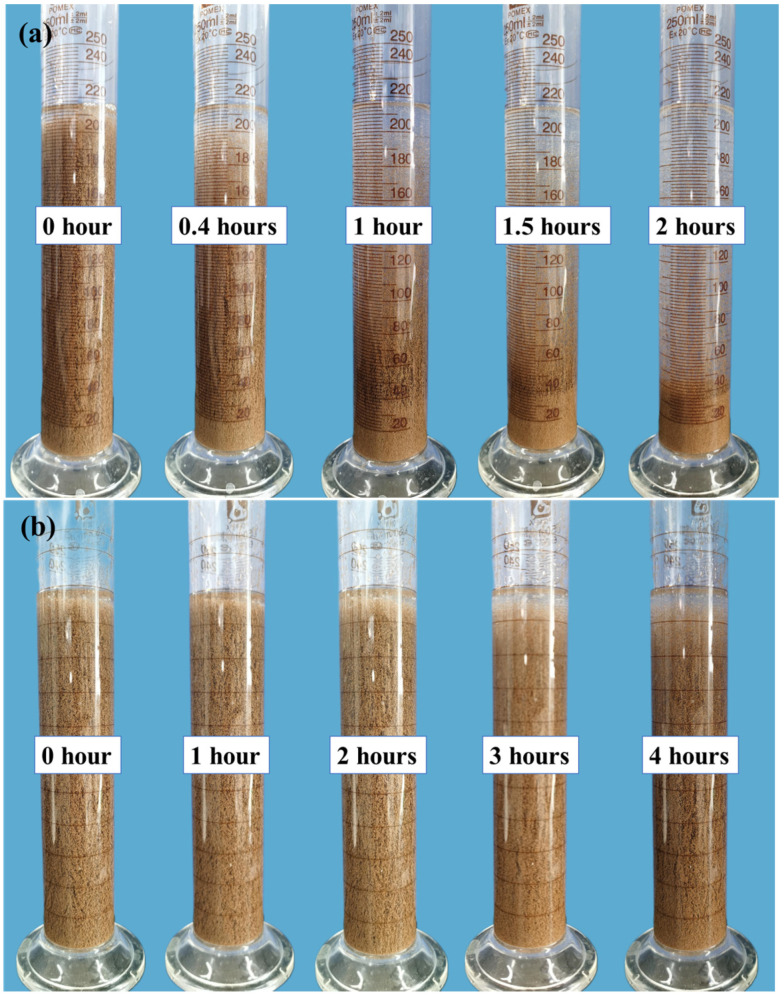
Static proppant-carrying capacity of the weighted fracturing fluid (0.4 wt% ASDM and 0.3 wt% crosslinker) at (**a**) 25 °C and (**b**) 90 °C.

The ability to transport proppants is a crucial characteristic of the weighted gel fracturing fluid. A greater proppant-carrying performance enables more proppant to be delivered into narrow fracture networks. The weighted fracturing fluid made with 0.4 wt% thickener and 0.3 wt% crosslinker is tested for proppant-carrying capacity at 25 °C and 90 °C, the results are displayed in Figure 3. The proppant settled entirely in 2 h at 25 °C. It was evident that no proppant settling occurred in the fluid at 90 °C. The settling rate at 90 °C was substantially lower than that at 25 °C, according to a comparison of the settling height of proppants during the same time period. The fracturing fluid started from an initial condition at 25 °C. In this stage, the zirconium ions present in the crosslinking agent were shielded by ligands, so they did not engage in crosslinking. The hydrophobic associating monomer in the thickener created a supramolecular thickener network and offered extensive non-covalent contacts even if the temperature of chemical crosslinking was not attained. Therefore, the system possessed a certain sand-carrying capacity [35]. At 90 °C, the carboxyl group on the thickener and the zirconium ions started to chemically crosslink, resulting in a more stable double network that induced a swift rise in viscosity as well as viscoelasticity. The formed dual-network structure has a higher strength and stronger sand-carrying capacity [36]. As a result, the weighted fracturing fluid had a good proppant-carrying capacity at high temperatures, making it more suitable for deep reservoir applications.

**Figure 4:**
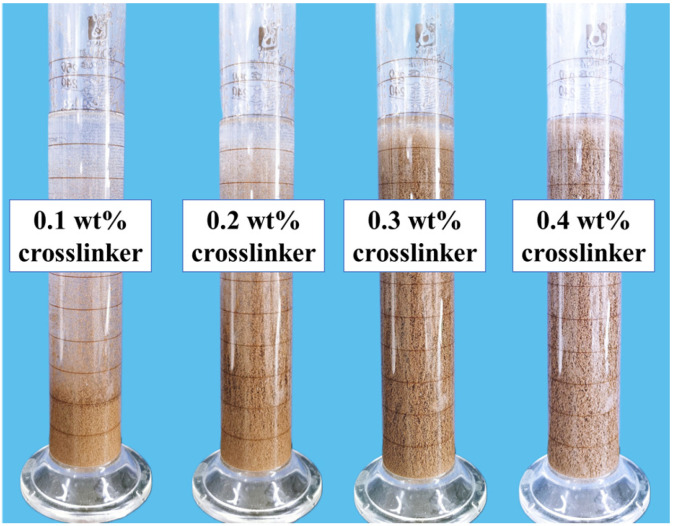
Dynamic proppant suspension ability of the weighted fracturing fluid as a function of crosslinker concentrations (0.1–0.4 wt%) after high-speed shearing at 90 °C for 4 h.

The fracturing fluid is subjected to 10,000 r/min shear for 5 s to simulate the high-speed shear forces experienced during passage through the perforation hole. The influence of the high-speed shear effect on the sand-carrying performance of the fracturing fluid is investigated, as shown in Figure 4. When the crosslinker dosage was 0.1 wt% and 0.2 wt%, the fracturing fluid was unable to effectively carry sand, resulting in sand loss. However, at crosslinker concentrations of 0.3 wt% and 0.4 wt%, the proppant did not settle during the 4 h static period of the fracturing fluid. Considering the static and dynamic proppant-carrying capacity of the fracturing fluid as it passed through the perforations under actual operating conditions, the crosslinker concentration in the fracturing fluid was set at 0.3 wt%.

### 2.4. Performance of Temperature and Shear Resistance

**Figure 5:**
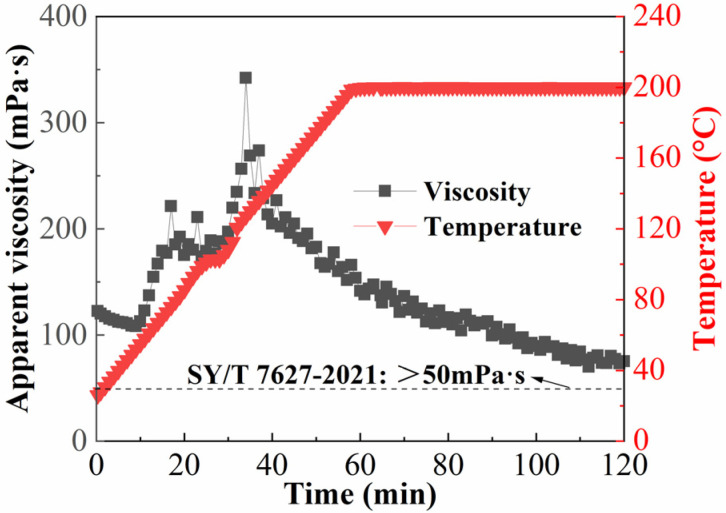
Temperature and shear resistance of the weighted fracturing fluid (0.4 wt% ASDM and 0.3 wt% crosslinker; HCOOK brine density 1.4 g/cm^3^) under a constant shear rate of 170 s^−1^. The temperature was ramped from 25 °C to 200 °C and then held at 200 °C for 120 min.

**Figure 6:**
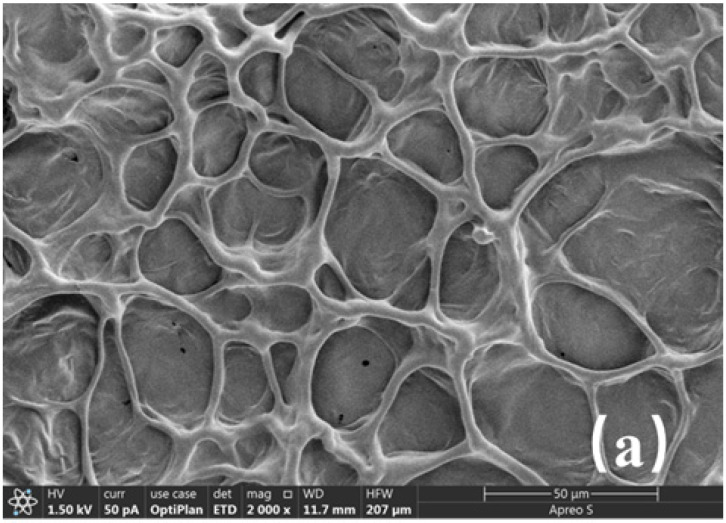

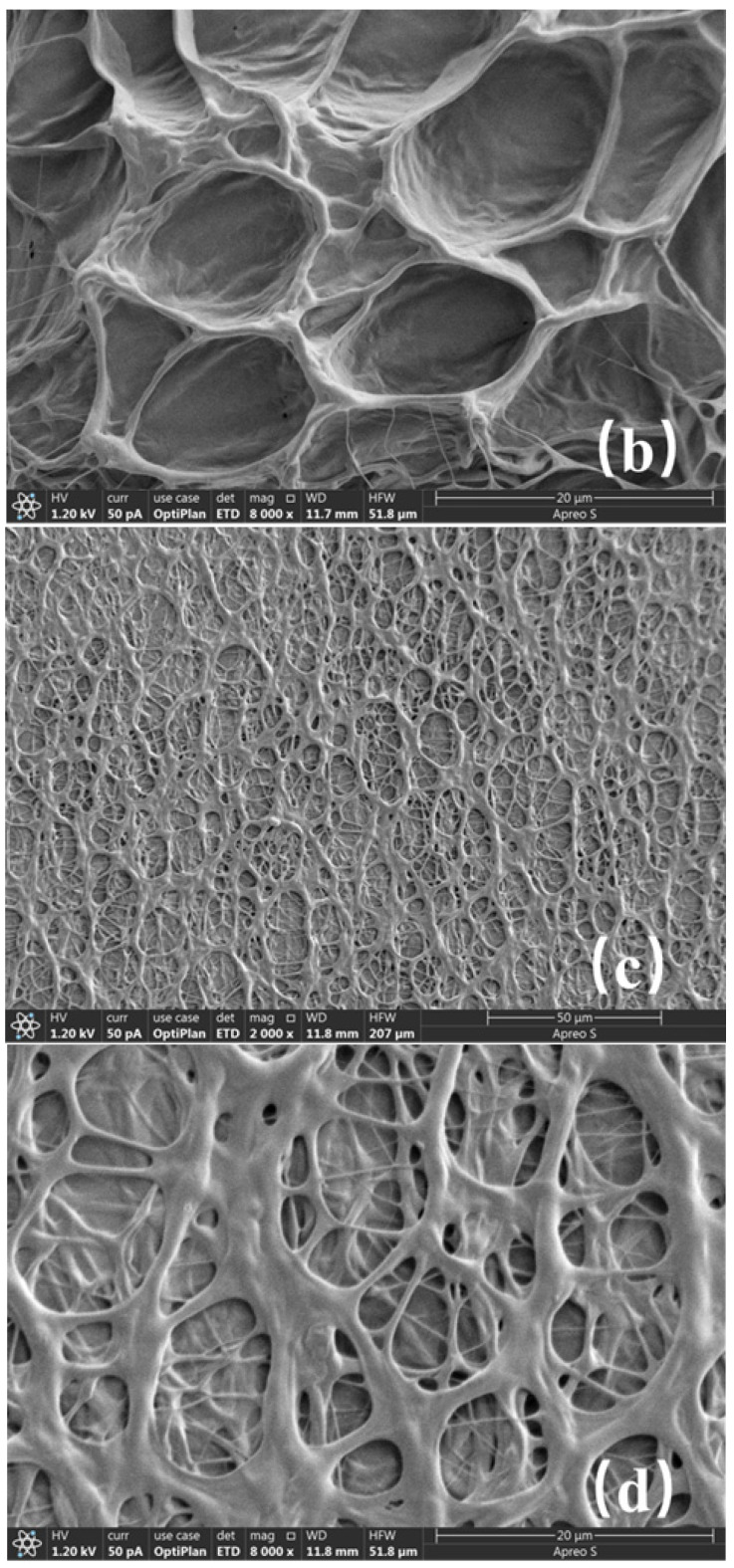
SEM images of the thickener-based fluid and weighted fracturing fluid. (**a**) 0.4 wt% thickener solution (magnification is 2000×), (**b**) 0.4 wt% thickener solution (magnification is 8000×), (**c**) the fracturing fluid made with 0.4 wt% thickener and 0.3 wt% crosslinker (magnification is 2000×), (**d**) the fracturing fluid made with 0.4 wt% thickener and 0.3 wt% crosslinker (magnification is 8000×).

Once the fracturing fluid enters the reservoir, it must withstand harsh conditions involving elevated temperatures and intense mechanical shear. Thus, the ability to withstand high temperature and shear is a key indicator for the fracturing fluid. Weighted fracturing fluid prepared by 0.4 wt% thickener and 0.3 wt% crosslinker was constantly sheared at 170 s^−1^ and 200 °C, the result is shown in Figure 5. Throughout the entire high-temperature constant shear process, the weighted fracturing fluid maintained a viscosity above 50 mPa·s, and the retained viscosity could reach 75.3 mPa·s following 120 min of shearing. This fully met the viscosity requirements of the hydraulic fracturing industry standards SY/T 7627-2021, “Technical Requirements of Water-Based Fracturing Fluid” for a fracturing fluid sand suspension [37]. It was shown that even in the presence of extreme heat and significant shear, weighted fracturing fluid maintained exceptional structural integrity. Furthermore, it was important to note that in Figure 6, the system’s viscosity essentially stayed the same at temperatures lower than 60 °C. Following that, a sharp rise in system viscosity was observed with increasing temperature and then showed a fluctuating decreasing trend, which was ascribed to the in situ reaction of the thickener’s carboxyl moieties with primary complexed zirconium ions, thereby establishing a crosslinked network under high-temperature conditions [38]. When the temperature was raised over 120 °C, there was only an obvious rise in viscosity, followed by a transient reduction. This phenomenon can be explained by the secondary complexed zirconium ions undergoing crosslinking, which produced a secondary network structure [28]. The results indicated that the weighted fracturing fluid had outstanding and consistent delayed crosslinking capabilities. The fracturing fluid successfully retained its low viscosity at low temperatures, enabling a reduction in wellbore friction, and the system’s viscosity gradually increased as a result of delayed crosslinking, allowing it to carry sand during the ultra-high temperature stage while maintaining good temperature and shear resistance performance.

### 2.5. SEM

The microstructure of thickener-based fluid and weighted fracturing fluid was observed by SEM images. As illustrated in Figure 6, the microstructure of thickener solution displayed a linear “honeycomb” distribution and interconnected molecular chains (Figure 6a,b). Due to the presence of supramolecular-associated groups, the thickener was able to accomplish several non-covalent supramolecular interactions, such as hydrogen bonding and hydrophobic interactions, which produced an ordered spatial network structure [39]. The double network created by the supramolecular thickener network and the crosslinked covalent thickener network in weighted fracturing fluid allowed for the observation of a dense “honeycomb” structure (Figure 6c,d). In a thickener-based fluid, the thickener network chain, which was subject to deformation and elastic energy release throughout the flow process, partially stored the kinetic energy produced by the fluid flow [34]. This prevented turbulence from developing, decreased energy loss, and decreased resistance. It could efficiently transfer energy and stress and keep it from settling when it was carrying proppant. Consequently, the low viscosity of the system contributed to lowering wellbore friction. For weighted fracturing fluid, a more uniform and denser dual-network improved the fluid’s ability to create a three-dimensional network structure and further enhanced the elasticity and stability. Consequently, the system exhibited high viscosity, which provided temperature resistance in the reservoir.

### 2.6. Gel Breaking Property

**Figure 7:**
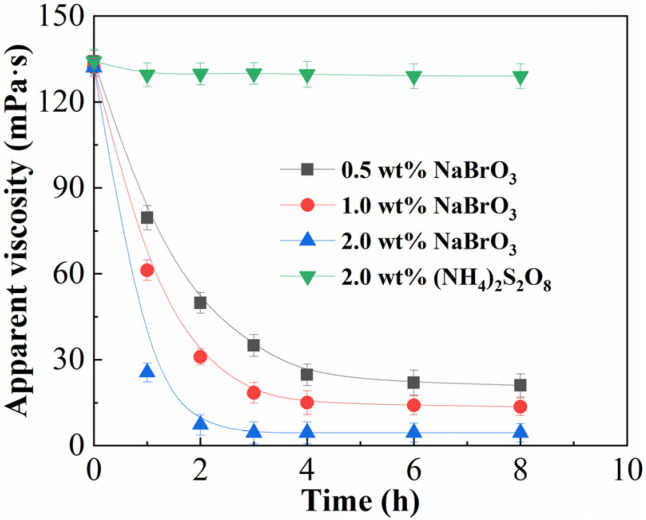
Apparent viscosity against gel-breaking time of weighted fracturing fluid (0.4 wt% ASDM and 0.3 wt% crosslinker) at 200 °C, comparing different breakers and concentrations: 0.5 wt% NaBrO_3_, 1.0 wt% NaBrO_3_, 2.0 wt% NaBrO_3_, and 2.0 wt% (NH_4_)_2_S_2_O_8_.

After reservoir fracturing is completed, the fracturing fluid needs to be returned to the ground as soon as possible to minimize damage to the reservoir. CHOOK is utilized as the weighing agent in this work; formate is reducible and readily oxidized by the oxidant, which can compete with the thickener for the gel breaker, making it difficult to break HCOOK-weighted gels with conventional breakers such as ammonium persulfate. This observation has been documented in previous studies. It demonstrates that the commonly used gel breaker (NH_4_)_2_S_2_O_8_ cannot break the CHOOK weighted fracturing fluid. Instead, NaBrO_3_, an inorganic salt with comparatively poor oxidation, is used to break the CHOOK weighted fracturing fluid at 200 °C. The experimental results are presented in Figure 7. The final viscosities of the gel-breaking fluid were measured at 21.5, 13.8, and 4.6 mPa·s after 8 h for 0.5 wt%, 1.0 wt%, and 2.0 wt% NaBrO_3_, respectively. The viscosity after 8 h at 200 °C remained above 100 mPa·s at 2.0 wt% (NH_4_)_2_S_2_O_8_, the commonly used gel breaker (NH_4_)_2_S_2_O_8_ is ineffective for HCOOK-weighted fracturing fluids. This implied that the gel structure of the CHOOK weighted fracturing fluid could only be successfully broken by a high concentration of the gel breaker. In the presence of NaBrO_3_, fracturing fluid degradation occurred when bromate groups oxidized the neighboring α-C-H bonds of thickeners, resulting in the production of a 1,2-diol-like structure [24]. Under elevated temperatures, this structure underwent homolytic cleavage, resulting in the degradation of the fracturing fluid. Since each molecular chain had tens of thousands of repeating units, the probability of the formation of a 1,2-diol-like structure remained relatively low [18]. Therefore, to enhance the production of this structure, a greater concentration of NaBrO_3_ was required. According to the industry standard SY/T 7627-2021, the gel-breaking fluid is required to exhibit a viscosity below 5 mPa·s. Consequently, 2.0 wt% NaBrO_3_ was chosen as the gel breaker concentration [37].

### 2.7. Mechanism Analysis of “Friction Reduction in Wellbore” and “Carrying Proppant in Reservoir” of Weighted Fracturing Fluids

**Figure 8:**
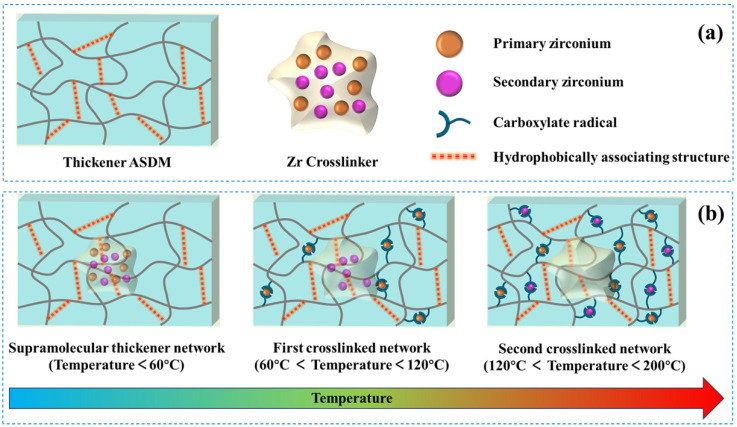
Schematic illustration of the supramolecular thickener network (below 60 °C) and the double crosslinked thickener network (above 60 °C). (**a**) Schematic diagram of thickener and crosslinker; (**b**) Mechanism of the tertiary release cross-linking strategy.

During injection of the fracturing fluid from surface pipelines into the reservoir, its network structure changes with temperature, as shown in Figure 8. When the temperature is lower than 60 °C, thickeners do not undergo crosslinking reactions with crosslinking agents; thickener chains of diverse morphologies become interwoven both vertically and horizontally, leading to chain entanglement. The system is dominated by a supramolecular thickener network. The network structure exhibits a certain degree of viscoelasticity; a portion of the kinetic energy generated by fluid motion becomes stored within the thickener network chain, which undergoes deformation and subsequently releases elastic energy as the fluid flows, preventing turbulence from developing, lowering resistance, and reducing energy loss, demonstrating excellent friction-reducing capability. At the same time, it can transfer energy and stress efficiently and keep it from settling when it is carrying proppant [40]. At temperatures higher than 60 °C, the primary complexed zirconium ions are progressively released; these ions then react with the thickener’s carboxylate moieties to form the initial crosslinked network, which raises the viscosity of the solution. The secondary complexed zirconium ions then crosslink to create an in situ second chemically crosslinked network when the system reaches the target reservoir, which is high temperature conditions. Consequently, the physically crosslinked network serves as a foundation for a subsequent increase in solution viscosity. Due to the combined effects of chemical and physical crosslinking, the double crosslinked polymer gel exhibits exceptional heat and shear resistance, a high viscosity at extremely high temperatures, and the ability to carry proppants in reservoir fractures at extremely high temperatures. This approach successfully keeps the fracturing fluid’s viscosity low at low temperatures to reduce friction in the wellbore, and the system’s viscosity gradually increases due to delayed crosslinking, enabling it to carry sand during the ultra-high temperature stage while retaining good temperature and shear resistance performance.

## 3. Conclusions

This work develops and evaluates an HCOOK-weighted fracturing fluid with delayed crosslinking capabilities that can be used in ultra-deep reservoirs at high temperatures and high pressures. It is possible to make the following conclusions.

(1) The weighted fracturing fluid possesses a density equal to 1.4 g/cm^3^ and is suited for ultradeep reservoirs with temperatures up to 200 °C, with the crosslinking process finished in 300 s at 90 °C.

(2) A maximum friction reduction rate of 63.8% is demonstrated by the weighted fracturing fluid. The friction reduction rate of fracturing fluid can reach 51.5% at 0.4 wt% thickener concentration, above the industry requirement of 50%.

(3) The weighted gel fracturing fluid made with 0.3 wt% crosslinker and 0.4 wt% thickener has a maintained viscosity of 75.3 mPa·s at 200 °C and 170 s^−1^. Additionally, the weighted gel experiences a secondary crosslinking process as a result of the greater temperature, improving the fracturing fluid’s ability to carry proppants at high temperatures.

(4) Complete gel breaking of weighted fracturing fluid is achieved by NaBrO_3_ at a concentration of 2.0 wt% under 200 °C for 8 h.

(5) Numerous hydrophobic associating groups in the thickener allow for multiple supramolecular interactions that build the non-covalent polymer network. The supramolecular interactions in the thickener enable the formation of a non-covalent polymer network, and the combination with an organic zirconium crosslinker produces a double crosslinked polymer network with delayed crosslinking behavior. The delayed crosslinked fracturing fluid is highly promising for ultra-deep hydrocarbon development, given its ability to simultaneously satisfy the need for low viscosity to minimize wellbore drag and high viscosity to resist elevated temperatures in the reservoir.

## 4. Materials and Methods

### 4.1. Materials

**Figure 9:**
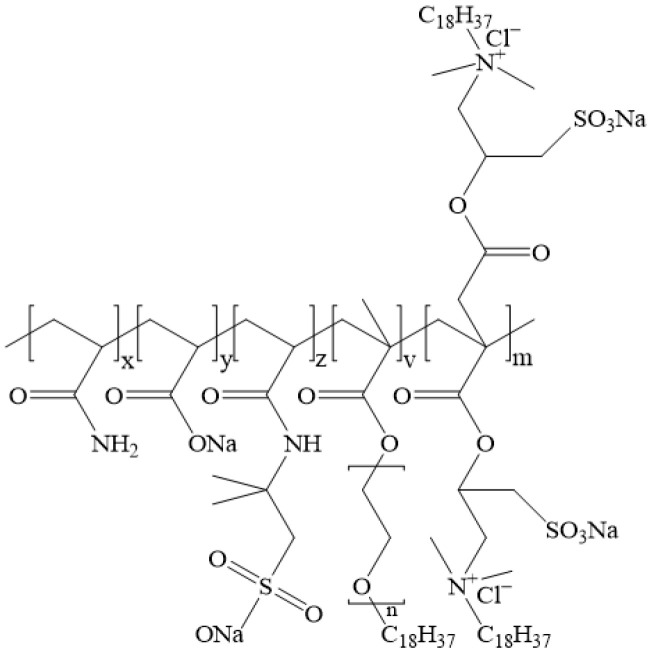
Chemical composition of the thickener ASDM.

Potassium formate (HCOOK, AR 98%) and sodium bromate (NaBrO_3_, AR 98%) were purchased from Shanghai Aladdin BioChem Technology Co., Ltd. (Shanghai, China). The hydrophobically associating thickener (ASDM, Figure 9) [22] and organic zirconium crosslinker (Zr-LSE) [28] were prepared according to previously reported methods. The chemical structure of the thickener with a viscosity-average molecular weight of 1.46 × 10^6^ g/mol was characterized (Figure 9). The ceramsite proppants (40/70 mesh, apparent density 2.45 g/cm^3^) were obtained from Jingang New Materials Co., Ltd. (Zouping, China). Deionized (DI) water was produced using a water purification system. All chemicals were used as received and were not further purified.

### 4.2. Sample Preparation

The preparation of the weighted gel fracturing fluid is based on Chinese Industry Standard SY/T7627-2021 “Technical Requirements of Water-Based Fracturing Fluid” [37]. The brine is prepared with an appropriate amount of HCOOK with a density of 1.4 g/cm^3^, which is used for preparing thickener solutions and fracturing fluids in this paper. According to our measurements and literature data, dissolving approximately 1500 g of HCOOK in 1 L of deionized water. First, a certain amount of HCOOK is added to the stirrer, and the speed is set to 600 r/min. Then, a certain amount of ASDM is dissolved in aqueous solution, and the mixture is stirred slowly and continuously until no solid thickener remains. Then, incorporation of the crosslinker into the thickener solution yields the weighted fracturing fluid.

### 4.3. Crosslinking Time

The delayed crosslinking behavior of the weighted fracturing fluid is evaluated by measuring its crosslinking time. First, 200 mL of thickener solution with HCOOK is prepared in a beaker, and then different concentrations of the crosslinker are added. Then, a glass rod is used to stir the mixture briskly while the timer begins. Crosslinking time is defined as the time elapsed from the addition of the crosslinker to the thickener solution until the fluid forms a gel that can be hung. The crosslinking time of fracturing fluids at 90 °C is tested. According to “SY/T 7627-2021”, the acceptable time range for crosslinking is 60–400 s [37].

### 4.4. Apparent Viscosity

Apparent viscosity measurements were conducted at 25 °C and a shear rate of 170 s^−1^ using a HAAKE MARS 40 rheometer (HAAKE, Karlsruhe, Germany) equipped with a Z43 cup (43 mm diameter) and a CC41 rotor (41 mm diameter).

### 4.5. Viscoelasticity

The viscoelastic modulus of the thickener solutions and fracturing fluids is measured by the MCR302 rheometer (Anton Paar, Graz, Austria) with a cone plate geometry CP50-1 (50 mm/1°) with a gap of 0.102 mm at 25 °C. Oscillation mode was employed for strain scanning, using a frequency of 1 Hz and a strain range of 0.1–1000%. The modulus of the fluid was defined as that measured in the linear viscoelastic region, where the modulus stays relatively constant.

### 4.6. Friction Reduction Performance

Friction reduction tests were conducted using a self-made loop drag test system that integrated three subsystems: a pumping system, a pipeline test system, and a data acquisition system. The fracturing fluid’s friction reduction performance at 25 °C was analyzed with a 3.3 m long pipeline having a diameter of 10 mm. Next, add simulated brine to the liquid distribution tank, open the circulating pump, adjust the flow rate, and wait for the pressure stability data collection system to automatically collect the pressure difference Δ*P*_1_, which is set as a blank reference. After passing through the pipeline at the same flow rate, the pressure drops Δ*P*_2_ of fracturing fluid were then measured, and Equation (1) is used to determine the friction reduction rate (error margin is 1%).

(1) $$DR\%=\frac{\Delta P_{1}-\Delta P_{2}}{\Delta P_{1}}\times 100\%$$

Here, *DR* is the friction reduction rate (%), Δ*P*_1_ is the pressure drop generated in brine (kPa), and Δ*P*_2_ is the pressure drop measured in fracturing fluid (kPa).

### 4.7. Temperature and Shear Resistance Performance

A HAAKE MARS 40 rheometer was used to evaluate the fracturing fluid’s temperature and shear resistance performance (with an error margin of 1.5%) over a temperature sweep from 25 °C to 200 °C. The measurement was conducted at a constant shear rate of 170 s^−1^ for 120 min once the target temperature was reached. The rheometer was equipped with a high-pressure sealed concentric cylinder and rotor assembly (PZ38 b), which required a sample volume of 32 mL.

### 4.8. Sand-Carrying Performance

Ceramsite particles were mixed with the weighted fracturing fluid at a sand ratio of 20%, and the mixture was stirred for 5 min. The resulting suspension was then poured into a glass cylinder, which was subsequently set inside a graduated cylinder. The experimental temperatures are 25 °C and 90 °C, respectively. During the static proppant suspension experiment, photographs were taken at one-hour intervals to document the proppant settling distance inside the measuring cylinder. The proppant settling rate was then defined as the ratio of the sand suspension liquid level’s settlement distance to the elapsed settling time. The prepared fracturing fluid is subjected to shear mixing at 10,000 r/min for 5 s using a Waring blender 8010S (Waring, Torrington, CT, USA). The aforementioned experimental procedure is repeated to observe the sand-carrying capacity of the fracturing fluid at 90 °C.

### 4.9. Microstructure Characterization

The fracturing fluid was first quickly frozen using liquid nitrogen, followed by vacuum freeze-drying to allow sublimation. The resulting frozen sample surface was then gold-coated. Morphological observations were carried out on an Apreo S SEM (Thermo Scientific, Waltham, MA, USA) at an accelerating voltage of 2.0 kV. While rapid freezing in liquid nitrogen is a common method for preserving hydrated polymer networks for SEM, we acknowledge that this process may cause some contraction of polymer chains. To minimize artifacts, the samples were first equilibrated at room temperature, then frozen rapidly, and freeze-dried immediately. The observed honeycomb structures are consistent with those reported in the literature for similar double-network hydrogels [6].

### 4.10. Gel Breaking Performance Test

About 200 mL of the weighed fracturing fluid is prepared, and different concentrations of gel breakers are added. Then, the weighed fracturing fluid is poured into the sealed container when the gel breaker is stirred well. Finally, the sealed container is put in the 200 °C ovens for a period of time to obtain the gel-breaking fluid. The gel breaking time is defined as the duration necessary for the fluid to reach a viscosity under 5 mPa·s.

## Data Availability

The data presented in this study are available on request from the corresponding author.
